# Proximity-based solutions for optimizing autism spectrum disorder treatment: integrating clinical and process data for personalized care

**DOI:** 10.3389/fpsyt.2024.1512818

**Published:** 2025-01-22

**Authors:** Annarita Vignapiano, Francesco Monaco, Stefania Landi, Luca Steardo, Carlo Mancuso, Claudio Pagano, Gianvito Petrillo, Alessandra Marenna, Martina Piacente, Stefano Leo, Carminia Marina Ingenito, Rossella Bonifacio, Benedetta Di Gruttola, Marco Solmi, Maria Pontillo, Giorgio Di Lorenzo, Alessio Fasano, Giulio Corrivetti

**Affiliations:** ^1^ Department of Mental Health, Azienda Sanitaria Locale Salerno, Salerno, Italy; ^2^ European Biomedical Research Institute of Salerno (EBRIS), Salerno, Italy; ^3^ Department of Health Sciences, University Magna Graecia of Catanzaro, Catanzaro, Italy; ^4^ Innovation Technology e Sviluppo (I.T.Svil), Salerno, Italy; ^5^ Department of Psychiatry, Faculty of Medicine, University of Ottawa, Ottawa, ON, Canada; ^6^ On Track: The Champlain First Episode Psychosis Program, Department of Mental Health, The Ottawa Hospital, Ottawa, ON, Canada; ^7^ Clinical Epidemiology Program, Ottawa Hospital Research Institute (OHRI), Ottawa, ON, Canada; ^8^ School of Epidemiology and Public Health, Faculty of Medicine, University of Ottawa, Ottawa, ON, Canada; ^9^ Childhood and Adolescent Neuropsychiatry Unit, Department of Neuroscience, Bambino Gesù Children’s Hospital (IRCCS), Rome, Italy; ^10^ Department of Systems Medicine, University of Rome Tor Vergata, Rome, Italy; ^11^ IRCCS Fondazione Santa Lucia, Rome, Italy; ^12^ Division of Pediatric Gastroenterology and Nutrition, Department of Pediatrics, Massachusetts General Hospital for Children, Harvard Medical School, Boston, MA, United States; ^13^ Mucosal Immunology Laboratory, Massachusetts General Hospital, Harvard Medical School, Boston, MA, United States

**Keywords:** autism spectrum disorder, artificial intelligence, machine learning, deep learning, patient-centered care

## Abstract

Autism Spectrum Disorder (ASD) affects millions of individuals worldwide, presenting challenges in social communication, repetitive behaviors, and sensory processing. Despite its prevalence, diagnosis can be lengthy, and access to appropriate treatment varies greatly. This project utilizes the power of Artificial Intelligence (AI), particularly Machine Learning (ML) and Deep Learning (DL), to improve Autism Spectrum Disorder diagnosis and treatment. A central data hub, the Master Data Plan (MDP), will aggregate and analyze information from diverse sources, feeding AI algorithms that can identify risk factors for ASD, personalize treatment plans based on individual needs, and even predict potential relapses. Furthermore, the project incorporates a patient-facing chatbot to provide information and support. By integrating patient data, empowering individuals with ASD, and supporting healthcare professionals, this platform aims to transform care accessibility, personalize treatment approaches, and optimize the entire care journey. Rigorous data governance measures will ensure ethical and secure data management. This project will improve access to care, personalize treatments for better outcomes, shorten wait times, boost patient involvement, and raise ASD awareness, leading to better resource allocation. This project marks a transformative shift toward data-driven, patient-centred ASD care in Italy. This platform enhances treatment outcomes for individuals with ASD and provides a scalable model for integrating AI into mental health, establishing a new benchmark for personalized patient care. Through AI integration and collaborative efforts, it aims to redefine mental healthcare standards, enhancing the well-being for individuals with ASD.

## 1 Introduction

Autism Spectrum Disorder (ASD) is a complex neurodevelopmental condition characterized by persistent difficulties in social communication and repetitive behaviors. According to the Diagnostic and Statistical Manual of Mental Disorders (DSM-5) (1), core features of ASD include challenges in social communication and interaction, such as limited eye contact, difficulty responding to verbal cues, and trouble with conversation flow. They may also display repetitive behaviors, like echolalia or fixated interests, and struggle with transitions or sensory sensitivities. Individuals with ASD may experience sleep disturbances and irritability but often exhibit strengths in detail-oriented learning, excelling in fields like math, science, music, and art. Despite the challenges associated with ASD, individuals with this condition exhibit unique strengths and abilities (2, 3). Individuals with ASD may prefer solitude, display limited interest in others, and struggle with physical affection (4). Autism is known as a “spectrum” disorder because there is wide variation in the type and severity of symptoms people experience. In DSM-5, the concept of a “spectrum” ASD diagnosis was created, combining the DSM-IV’s separate pervasive developmental disorder (PDD) diagnoses: autistic disorder, Asperger’s disorder, childhood disintegrative disorder, and pervasive developmental disorder not otherwise specified (PDD-NOS), into one. People of all genders, races, ethnicities, and economic backgrounds can be diagnosed with ASD. However, ASD can be a lifelong disorder. The worldwide prevalence comprised 72 per 10,000 for Autism spectrum disorder, 25 for Autistic Disorder, 13 for Asperger Syndrome, and 18 for the combined group of Atypical Autism and Pervasive Developmental Disorder- Not Otherwise Specified (5). In Italy, ASD prevalence is estimated at 13.4 per 1,000 children aged 7–9 years, with a male-to-female ratio of 4.4:1 (6). The etiology of ASD is multifactorial, involving both genetic and environmental influences. Siblings of individuals with ASD have a higher risk, suggesting a strong genetic component (7). Environmental factors, such as prenatal medication exposure and advanced parental age, may also contribute (8). The analysis by Janet Cakir et al. (9) examines the lifetime social costs of ASD in the United States from 1990 to 2029. ASD incurs a staggering estimated lifetime social cost averaging around $3.6 million per affected individual. The cumulative burden of these costs has already surpassed $7 trillion, roughly equivalent to two years of total federal revenue for the nation. If the prevalence of ASD remains steady, the projected cost by 2029 is estimated to reach $11.5 trillion. However, if prevalence rates continue their current upward trajectory, the projected costs could skyrocket to nearly $15 trillion. In Italy, Micai et al. (10) show that the care of people with ASD leads to caregiver productivity loss, employment difficulties for autistic adults, and impacts on mental well-being and overall quality of life.Individuals with Autism Spectrum Disorder (ASD) frequently encounter a range of medical and psychiatric comorbidities that significantly impact their well-being. Common medical issues include gastrointestinal problems, which can manifest as chronic constipation, diarrhea, or abdominal pain, and epilepsy, which affects a substantial subset of individuals with ASD (11). These medical conditions can complicate the daily lives of those with ASD, contributing to discomfort and additional health concerns. On the psychiatric front, anxiety and depressive disorders are prevalent among individuals with ASD. Anxiety can manifest in various forms, such as social anxiety, generalized anxiety disorder, or specific phobias, making social interactions and daily activities challenging (12). Depressive disorders further exacerbate these difficulties, often leading to diminished motivation, increased irritability, and overall reduced quality of life (13). Attention Deficit Hyperactivity Disorder (ADHD) is another common comorbidity, characterized by inattention, hyperactivity, and impulsivity. The presence of ADHD in individuals with ASD can complicate the clinical picture, making it harder to differentiate between the symptoms of the two conditions and complicating treatment plans (14). Moreover, some individuals with ASD may experience psychosis, including symptoms such as hallucinations or delusions (15) with a rate of conversion to full psychosis (16). The co-occurrence of psychosis can lead to significant functional impairment and necessitates specialized psychiatric care (17). These comorbidities complicate the diagnosis and treatment of ASD, as the overlapping symptoms can obscure the clinical picture and require a nuanced approach to care. The presence of multiple comorbid conditions necessitates comprehensive, multidisciplinary care involving specialists from various fields, including gastroenterology, neurology, psychiatry, and psychology, to address the diverse needs of individuals with ASD effectively. This holistic approach aims to improve the overall quality of life for patients by managing each comorbid condition and supporting the individual’s developmental and emotional needs. Early intervention is paramount in addressing the unique needs of individuals with ASD, as it can facilitate the development of crucial social skills and behavior management strategies. However, ASD remains a lifelong condition, significantly impacting daily functioning and social interactions, with implications for the well-being of both individuals and their caregivers. Advancements in technology, particularly AI, ML, and DL, promise to enhance ASD care through remote monitoring and personalized interventions (18–20) These innovations offer opportunities to optimize treatment delivery and improve outcomes for individuals with ASD (21, 22).

In alignment with this vision, we have identified a range of AI models that will be systematically evaluated for their potential to enhance the diagnostic and therapeutic capabilities of the MDP. Among the models considered, ClinicalBERT (23) demonstrates strong potential in processing both structured and unstructured patient records through its advanced text interpretation capabilities. Similarly, Ada (24), with its focus on mental health applications, is particularly relevant for adaptive assessments and improving patient engagement in clinical settings. Additionally, AgentClinic (25) offers promising prospects for task-specific fine-tuning, which could prove invaluable in predicting ASD-related outcomes. The multimodal nature of CLIP provides unique opportunities to analyze non-verbal cues, a critical component in ASD diagnosis. To ensure a comprehensive approach, we will also examine insights from the review by Mehandru et al. (26), which highlights advanced tools and methodologies for clinical integration. This exploration aims to identify adaptable, scalable solutions that align seamlessly with the objectives and infrastructure of the Master Data Plan MDP.

In Italy, individualized treatment options for individuals with ASD remain scarce, with significant gaps in the healthcare system contributing to barriers in accessing specialized care. Many families face prolonged waiting periods for diagnosis, which often delays early intervention, a crucial factor in improving long-term outcomes. Furthermore, there is a limited number of healthcare providers trained in ASD-specific therapies, particularly outside major cities, leaving many without the support they need. The MDP seeks to address these challenges by introducing innovative, AI-driven tools that can streamline diagnosis and provide personalized treatment plans, bridging the gap between demand and availability (10). This approach has the potential to make specialized ASD care more accessible, scalable, and tailored to individual needs, setting a new standard for mental health support across the country.

Similar initiatives in other countries have demonstrated the feasibility and impact of AI- driven ASD care. For example, platforms like the UK’s SCAMP system and Canada’s Autism Navigator have shown significant improvements in early intervention and resource allocation. Comparing the MDP with these systems highlights its unique approach to personalized care through real-time predictive analytics and caregiver engagement. We conducted an in-depth analysis of the AI models considered for integration into the MDP. Moreover, all the above models, despite their strengths, present certain limitations.

ClinicalBERT (23), for instance, requires significant fine-tuning to adapt to ASD-specific language. Ada, while effective in mental health applications, may not fully address the complexity of ASD comorbidities (24). AgentClinic’s fine-tuning processes necessitate large, annotated datasets, which are often unavailable for ASD populations (25). CLIP, though powerful in multimodal analysis, has limited specificity for clinical settings (27). Given these constraints, we decided to develop a tailored AI solution that aligns more closely with the unique requirements of ASD diagnosis and management. Our custom model incorporates domain-specific datasets and prioritizes seamless integration with the MDP’s architecture, ensuring optimal performance and usability in real-world clinical applications. This approach allows us to overcome the limitations of existing models while creating a scalable, precise, and efficient tool for ASD care.

In alignment with the National Action Plan for Mental Health (NAPMH) guidelines, the Local Health Unit of Salerno’s Department of Mental Health strategically plans to enhance therapeutic services for individuals with ASD. The overarching objective is to address the increased psychological distress experienced by individuals with ASD and their families, particularly exacerbated by the ongoing epidemic. The project aims to strengthen the support infrastructure for individuals with ASD and their families to effectively address the unique challenges posed by the current public health situation. To achieve these goals, the MDP focuses on the following key objectives:

Key objectives include:

1. Updating the mapping of all services in the territorial network dedicated to ASD.
2. Providing access functionality to care-taking archives, offering information on available services and individual status within their care setting.
3. Implementing a management and monitoring dashboard tailored to ASD care.
4. Establishing a system for receiving and sending notifications integrated with the transitions dashboard.
5. Ensuring privacy and data security in compliance with ASD-specific regulations.
6. Generating reporting and flow analysis relevant to ASD care services.

## 2 Materials and methods

**Figure 1:**
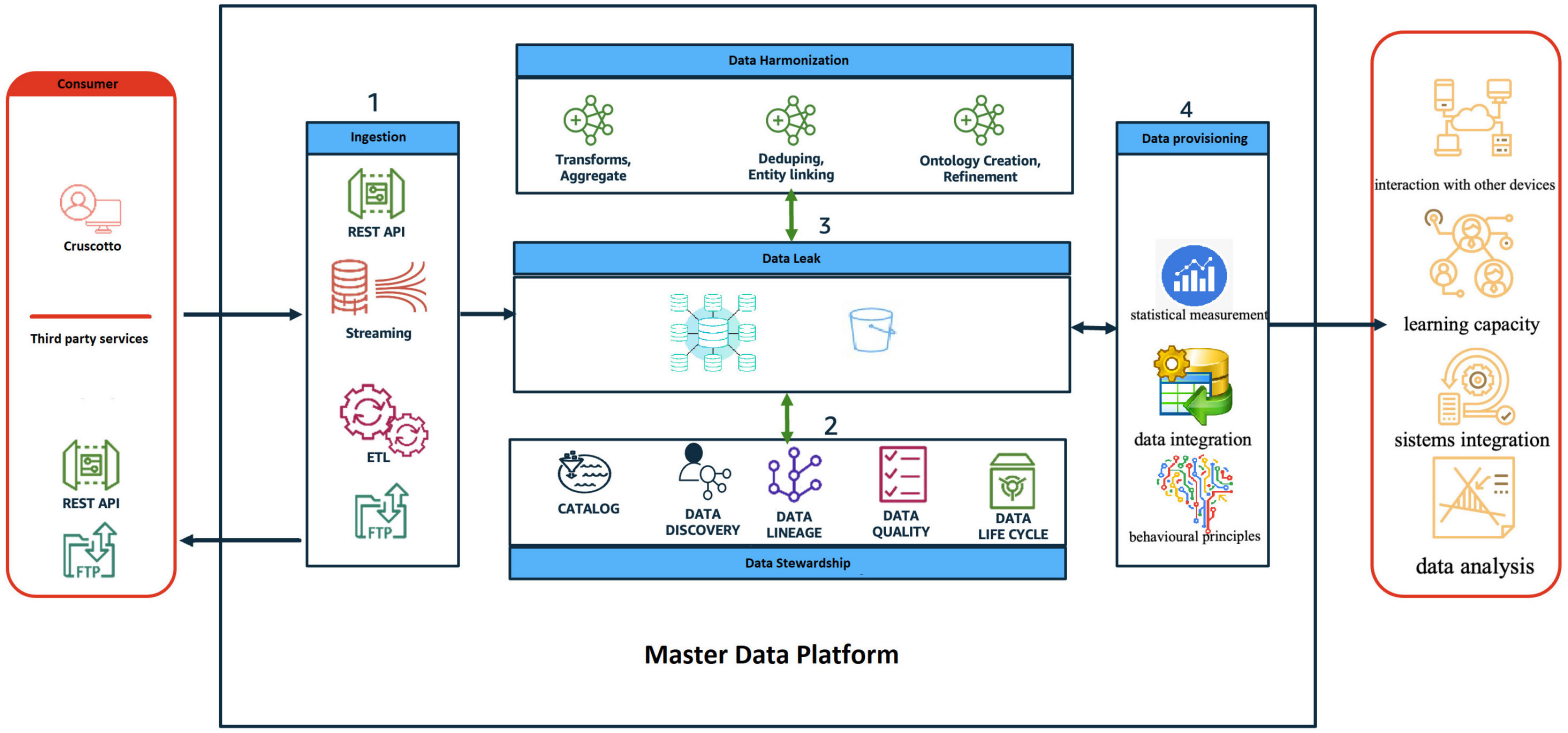
The diagram provides a high-level view of the components and how they interact through the different phases of the data life cycle.

In collaboration with IT.Svil s.r.l., we have developed the Master Data Platform (MDP) (**Figure 1**), a sophisticated data-driven ecosystem designed to fulfill our objectives. This innovative approach leverages an objective decision-making algorithm that significantly reduces reliance on subjective judgments that are often susceptible to errors. The same working group has successfully implemented a study protocol for an advanced Artificial Intelligence (AI) platform aimed at delivering personalized treatment for Eating Disorders (28). This platform is specifically designed to tailor treatment plans to individual patients, thereby potentially enhancing outcomes and lowering relapse rates. The involvement of the same team in both the development and implementation phases reinforces their strong commitment and expertise in this critical area, which could lead to more effective and efficient treatment options, as well as improved access to care for affected individuals.The MDP incorporates intelligent agents at various care stages. A pre-trained large language model (LLM) enhances patient and caregiver engagement (26), while an NLP-based chatbot gathers information via a mobile app, enriching the diagnostic process. Machine learning agents (Decision Trees, Random Forests) predict the likelihood of specific autism-related behaviors, and generative models (GANs, Variational Autoencoders) simulate treatment scenarios and generate personalized data representations for targeted therapy (29). The MDP facilitates real-time access and secure sharing of patient data across the hospital and university network, enhancing care pathway management and ensuring consistency. Its patient-centered approach empowers families to actively participate in care, track data, and engage in decision-making. Data analysis informs tailored treatment plans and interventions, ultimately improving the care experience. The scalable, interoperable architecture integrates with various regional and corporate healthcare systems using defined APIs and established healthcare standards (HL7/FHIR, HL7, HL7/CDA2) and secure communication protocols (HTTPS over SSL, SFTP, SCP, VPN). A diagram depicts the system’s components and their interactions.

The platform’s development builds on established machine learning best practices, such as robust cross-validation and upsampling to mitigate class imbalances, and the use of Gini importance and permutation tests to assess feature importance. While acknowledging limitations inherent in data collection (e.g., potential for multi-center variability), the MDP represents a significant advancement in ASD care management, offering the potential for improved diagnostic accuracy, personalized treatment plans, and more efficient resource allocation.

The real-time access and secure sharing of patient data, efficiently managing valuable information related to ASD across the hospital and university network in the Campania region. The MDP supports healthcare providers and serves as a vital resource for patients and their families, enhancing the management of care pathways to ensure consistency throughout treatment. By embracing a patient-centered approach, the MDP empowers families to actively participate in care, allowing them to track their loved ones’ data and engage in decision-making. The platform analyzes this data to generate insights that inform tailored treatment plans and interventions, ultimately fostering a more effective care experience for individuals with ASD and their families. The proposed architecture is designed as a scalable solution that allows for flexible and easily extendable systems, with a strong emphasis on interoperability. It can integrate with various regional and corporate healthcare systems, including patient registries and hospital admission management systems. Each system will have defined entry points through specific APIs (application programming interfaces) that ensure secure data exchange, governed by stringent data control policies and protection measures. Established healthcare standards, such as HL7/FHIR, HL7, and HL7/CDA2, will be utilized to facilitate interoperability, while secure communication protocols like HTTPS over SSL, SFTP, SCP, and VPN will ensure data transmission safety. The accompanying diagram provides a high-level overview of the system’s components and their interactions throughout the data lifecycle.

The role and behavior of each application layer is described below:

- Consumer: represents all the users of the MDP system both as an input system (i.e., health workers, patients/caregivers, etc) and as an output system (i.e., regional operators, patients/caregivers, health workers, etc);
- Ingestion: represents the frontier layer of MDP and has the role of servicing all the input requests coming from the consumer systems.
- Data Harmonisation (ensuring consistency between datasets from different sources): in this layer, the input data are “harmonized, i.e., all the data cleaning and/or augmentation techniques/procedures are applied where necessary.
- Data Stewardship: has the task of ‘administering’ quality, privacy and security data.
- Data Leak: represents the application layer in charge of data storage.
- Data Provisioning: provides tools/routines/programs to transform data to make it information, for example:

➔ ML algorithms to sperform predictive analysis;
➔ Generate reporting (BI);
➔ Producing data flows, e.g., in CSV format (e.g., health flows)

To integrate AI tools into MDP, we will examine and compare two primary training approaches: pre-training and fine-tuning. Pre-training from scratch will be considered for highly specialized models tailored to ASD-specific datasets while fine-tuning existing clinical AI tools with ASD-annotated datasets offers a practical balance of accuracy and resource efficiency. Both strategies will be rigorously assessed using key performance metrics, including precision, recall, and F1-score, to ensure clinical relevance and alignment with the platform’s objectives. Our action plan for integrating AI tools into the MDP includes several critical steps. First, we will conduct a systematic review of proposed models alongside additional options identified through literature review and expert consultations. Top candidates will undergo pilot testing in a controlled environment with de-identified patient datasets. Their performance will be analyzed against predefined clinical benchmarks to verify efficacy and relevance. After selecting the best tools, they will be implemented into the MDP, with ongoing monitoring of their impact on diagnostic accuracy, patient outcomes, and resource allocation to facilitate continuous improvement. To ensure the effective evaluation and selection of AI tools for MDP integration, we have established a robust framework based on key criteria. The guiding principle emphasizes that AI tools should enhance the MDP protocol rather than dictate its framework. Our selection and implementation processes will prioritize adaptability, ensuring compatibility with the existing MDP data pipeline and architecture; accuracy in predicting and interpreting ASD-related outcomes; and scalability to handle increasing data volumes and diverse user needs. Compliance with GDPR and data governance standards will also be prioritized, along with usability to ensure seamless integration and user-friendliness for clinicians and caregivers. This comprehensive approach will help align selected tools with the platform’s goals and practical requirements.

The platform endeavours to construct an IT infrastructure that embraces a comprehensive approach, not merely from a functional standpoint but also in user experience. It comprises various components, each with its objectives, yet collectively contributing to a unified purpose. For instance, leveraging technologies like mobile devices empower patients to actively engage in their treatment journey, revolutionizing therapeutic approaches. Simultaneously, healthcare professionals gain access to control tools fostering continuous and direct patient interaction. Patient information is safeguarded through comprehensive data security protocols designed to ensure privacy and regulatory compliance. All patient data is protected by end-to-end encryption, both during transmission and in storage, preventing unauthorized access at any stage. To further secure patient identities, data anonymization techniques are applied, ensuring that any identifying information is either removed or obscured to maintain confidentiality. Regular audits and security checks are conducted to ensure compliance with GDPR standards, which mandate strict guidelines on data handling, access controls, and storage. These measures collectively ensure that patient data remains private, secure, and accessible only to authorized personnel, providing both patients and practitioners with confidence in data protection.

### 2.1 Autistic symptoms assessment

Patients undergo thorough evaluations by pediatricians, neuropsychiatrists and psychologists to diagnose ASD in accordance with DSM-5 criteria. This process will incorporate gold-standard assessment tools such as the Autism Diagnostic Observation Schedule, 2nd Edition (ADOS-2), and the Autism Diagnostic Interview Revised (ADI-R) (30). The ADOS-2 is a semi-structured assessment focusing on communication, social interaction, and play, delivering calibrated severity scores with high reliability and specificity. The ADI-R consists of a caregiver interview addressing social behavior, communication, and repetitive behaviors, and is appropriate for individuals with a mental age of 18 months or older. Along with these tools, it is essential to adopt a balanced diagnostic approach that integrates standardized tools and clinical expertise among the various health professionals involved (31, 32). This includes evaluating diagnostic reliability through metrics such as Positive Predictive Value (PPV) and Negative Predictive Value (NPV), while tailoring individualized assessment plans to the unique characteristics of each patient (33). To ensure the reliability of AI-assisted annotations, we will adopt established agreement metrics to compare outputs with human evaluations. We plan to leverage Positive Predictive Value (PPV) and Negative Predictive Value (NPV) metrics, following the approach outlined by Penner et al. (33). These metrics will be used to calculate agreement values between pediatricians and specialized psychiatrists, providing a robust benchmark for validating annotations. By applying this methodology, we aim to compare the annotations made by medical doctors (MDs) with those generated by AI-assisted tools for ADOS and ADI-R metrics, ensuring consistency and reliability in the diagnostic and evaluative process. In addition to statistical metrics such as PPV and NPV, we will incorporate methodological frameworks to enhance validation processes. We will explore the cut-off approaches outlined by Lord (30), as these methodologies provide a robust framework for determining agreement scores and refining the validation of annotations across human evaluators and AI tools (34). To further enhance the robustness of the evaluation process, additional considerations will include we will consider additional agreement metrics and methodologies as needed throughout the development process, maintaining flexibility and adaptability within the evaluation framework. Cognitive development will be assessed using the Wechsler Intelligence Scale for Children (WISC-IV) (35), which provides various index scores. For individuals facing language challenges, non-verbal tests like the Leiter International Performance Scale–3rd Edition (Leiter-3) (36), Colored Progressive Matrices (CPM) (37), and the Griffiths Mental Development Scales—Extended Revised 0–2—GMDS-ER 2–8 (GMDS-ER) (38) will be employed to evaluate intelligence and developmental quotients, focusing exclusively on non-verbal scores. Adaptive functioning will be evaluated using the Vineland Adaptive Behavior Scales–Second Edition (VABS II) (39) and the Italian version of the Adaptive Behavior Assessment System-Second Edition Parent Form 0–5 and Form 5–21 (ABAS II) (40). The VABS II assesses adaptive capabilities through caregiver interviews, offering scores across communication, socialization, and daily living skills. The ABAS II, a parent-report questionnaire, generates domain scores in conceptual, social, and practical areas. Both instruments offer reliable composite scores and are widely used in ASD research.

## 3 Results

This project introduces an innovative MDP platform specifically designed for the management and treatment of ASD within a territorial psychiatric care setting. The platform promises numerous advantages for both patients and healthcare providers. At its core, the MDP aims to cultivate a more accessible and patient-centered environment. By engaging in interactive dialogues with a program chatbot and healthcare professionals, patients and caregivers are empowered to take an active role in their care pathway, potentially leading to improved adherence and clinical outcomes. Furthermore, the MDP intends to enhance healthcare delivery efficiency by streamlining processes such as waiting lists, resource allocation, and patient transitions between facilities. It is projected that implementing the MDP could reduce diagnostic wait times by up to 30%, facilitating quicker access to care for individuals with ASD. Transitioning from traditional paper records to readily accessible electronic health data allows attending physicians to gain a comprehensive view of each patient’s medical history, aiding in the tailoring of treatment plans to individual needs. By incorporating psychosocial variables, the platform further personalized treatment approaches, potentially enhancing treatment adherence, quality of life, and overall patient satisfaction. A key feature of the platform is its ability to streamline waiting list management, ensuring prompt access to care for individuals with ASD. By alleviating the delays and uncertainties associated with seeking treatment, the platform promotes a smoother and more efficient care experience. Additionally, MDP-based decision-making optimizes care intensity, enabling careful evaluations of admissions and transfers between facilities and aligning resource allocation with individual patient needs. This approach underscores a commitment to patient-centered care and resource efficiency.

Robust data governance is central to the platform’s design, safeguarding the ethical and secure management of sensitive patient information. This instills confidence in both patients and providers by establishing a high standard for responsible data practices within territorial mental healthcare. The integration of AI technology also holds great potential for enhancing diagnostic accuracy and treatment efficiency, with preliminary estimates suggesting that it could improve diagnostic accuracy rates by 20-25%, facilitating earlier intervention for patients. Moreover, technology serves as a vital ally in the treatment process, providing invaluable support to physicians and empowering patients in their recovery journey. By improving access to care for those in need, the platform contributes to a more equitable healthcare landscape. Its development and implementation foster optimized care pathways and enhanced data sharing among facilities, promoting collaboration and resource sharing across the healthcare system. Establishing a departmental hub ensures effective monitoring of patient clusters across all spoke facilities, fostering transparency and a cohesive approach to territorial mental healthcare.

Ultimately, the MDP transforms healthcare workflows by streamlining data sharing and enhancing clinical decision-making through real-time predictive analytics. By integrating patient data from various sources, the platform enables seamless communication between healthcare providers, reduces administrative burdens, and minimizes delays in accessing crucial patient information. This centralized data approach supports real-time decision-making, allowing clinicians to leverage predictive analytics to assess patient needs and anticipate potential issues, enabling early intervention. Additionally, MDP’s insights assist providers in personalizing treatment plans more efficiently, fostering a more collaborative, data-driven approach to patient care that ultimately improves outcomes and optimizes resource allocation within the healthcare system.

## 4 Discussion

A secondary analysis by Klaiman et al. (31) of a multi-site clinical trial involving young children referred to specialized ASD centers demonstrated that 29.8% of cases received either an ASD or Non-ASD diagnosis, accompanied by varying levels of clinician uncertainty. Mid-level autism-related symptoms, as measured by the ADOS-2, were identified as strong predictors of clinician uncertainty, increasing the risk of misdiagnosis or delayed access to treatment for children with uncertain clinical profiles. Given the alarmingly high prevalence of false negatives in ASD diagnoses, particularly among younger children, there is an urgent need for standardized quantitative protocols to improve diagnostic reliability and decision-making. While false positives can lead to unnecessary alarms, they can also facilitate access to beneficial interventions for children with developmental delays, highlighting the importance of understanding the costs and benefits of diagnostic decisions amid uncertainty. MDP represents an innovative approach to ASD management, marking a significant shift toward patient-centered care. By promoting interactive communication channels between patients, caregivers, and healthcare providers, MDP enables people to actively participate in the diagnostic and treatment pathway, potentially improving adherence and clinical outcomes. Furthermore, it aims to enhance the efficiency of healthcare delivery by streamlining processes such as waiting lists, resource allocation, and transitions between facilities, thereby reducing the burden on patients and optimizing resource utilization within the healthcare system. Utilizing advanced data analysis techniques, the MDP seeks to improve diagnostic accuracy and tailor treatment plans to the unique profiles of each patient. By facilitating data sharing and collaboration among different facilities, it promotes a cohesive approach to ASD care, ensuring consistent standards and enhanced service delivery across the entire territory. However, the implementation of the MDP brings forth important considerations regarding data security, user adoption, and integration with existing healthcare systems. Ensuring the ethical and secure handling of sensitive patient information is paramount, necessitating robust data governance measures such as encryption protocols and access controls. Encouraging active platform utilization among healthcare professionals, patients, and caregivers is essential for success, requiring targeted training programs and user-friendly interface design. Additionally, seamless integration with existing healthcare systems is crucial for widespread adoption, necessitating strategies that ensure interoperability through standardized data formats and APIs. Ultimately, a comprehensive, multidisciplinary approach to care is essential for addressing the diverse needs of individuals with ASD. This approach involves collaboration among specialists from various fields, including gastroenterology, neurology, psychiatry, and psychology. Such collaboration ensures that all aspects of the individual’s health are considered, leading to more effective management of comorbid conditions and ultimately enhancing the overall quality of life.

Future enhancements to the MDP could significantly expand its capabilities and impact. Integrating advanced AI functionalities, such as personalized learning modules and communication assistance tools tailored to the unique needs of individuals with ASD, holds significant promise. The complexity of ASD, often involving multiple comorbidities, necessitates a different approach to treatment (41); AI could aid in developing more precise diagnostic tools and personalized treatment plans that effectively address these comorbidities. Furthermore, expanding the platform’s scope to encompass other neurodevelopmental disorders would broaden its reach and impact, potentially offering similar benefits to individuals and families affected by a wider range of conditions. This would necessitate further research to develop specific algorithms and training data for each disorder.

**Figure 2:**
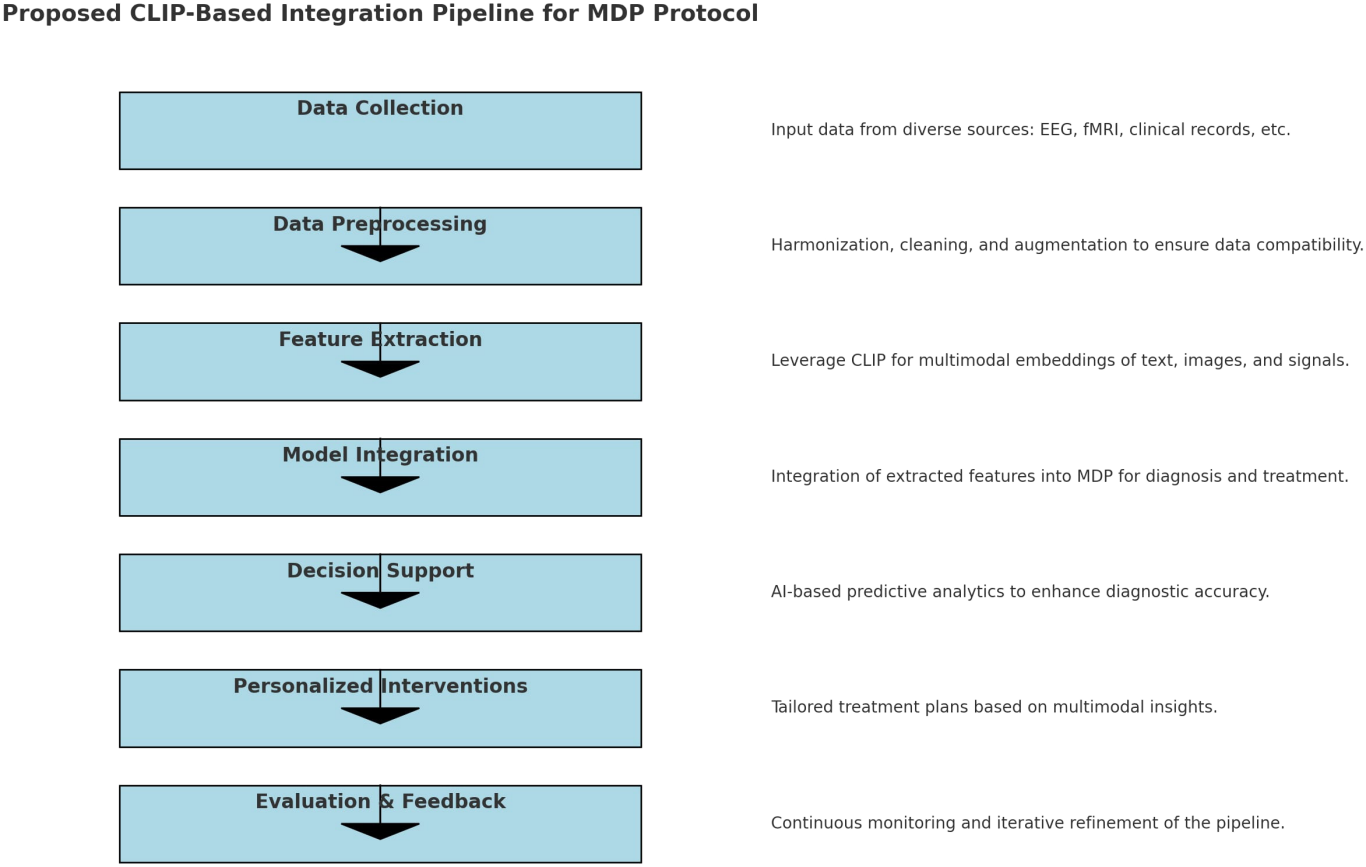
Proposed CLIP-Based Integration Pipeline for MDP Protocol.

Utilizing advanced data analysis techniques, the MDP seeks to improve diagnostic accuracy and tailor treatment plans to the unique profiles of each patient. Multimodal integration significantly improves accuracy in diagnosing and treating complex conditions like ASD by leveraging diverse data types such as EEG, fMRI, and clinical text. This approach enriches information, reduces ambiguity by cross-validating across modalities, and enhances feature representation through shared embedding spaces, as demonstrated by models like CLIP. For example, Neuro-GPT EEG (42) integrates EEG with text data for improved cognitive state predictions, while BrainCLIP fMRI (43) and MindEye2 (44) demonstrates the power of multimodal embeddings. MindEye2 uses a novel shared-subject model, a multimodal approach (incorporating a diffusion prior, retrieval, and low-level submodules), and a fine-tuned Stable Diffusion XL model to improve the accuracy and semantic richness of fMRI-based image reconstructions. The use of a shared latent space and OpenCLIP embeddings enhances performance. BrainCLIP fMRI’s alignment of neural signals with external stimuli highlights the benefits of multimodal embeddings, particularly for robust generalization, bias reduction, and personalized interventions crucial in applications like tailored ASD care (**Figure 2**).

This study protocol presents a groundbreaking platform tailored for the management of ASD while also highlighting several important limitations that warrant discussion.

Currently, the platform’s effectiveness lacks empirical validation, a gap to be addressed by a pilot study in Salerno. This pilot is crucial for a comprehensive cost-benefit analysis, essential for determining national-level viability within the Italian healthcare system. The pilot will also critically assess cost-effectiveness and user-friendliness, especially considering the varying levels of digital literacy among patients and caregivers.A significant concern is the platform’s reliance on AI algorithms. The ability of these algorithms to generalize beyond the training data and perform reliably across diverse patient populations needs thorough evaluation. The pilot study should assess the performance of the AI models on data from the Salerno Local Health Unit, and ideally from other units, to evaluate their generalizability. The risk of overfitting the models to the characteristics of the training data must be carefully considered and mitigated. Bias in training datasets presents a major ethical and practical concern. The representativeness of the training data with respect to the diversity of the ASD population (e.g., age, gender, socioeconomic status, severity of symptoms, comorbidities) needs rigorous scrutiny (45). The pilot study must collect and analyze data on potential biases in the training data. Strategies to address this will be developed and assessed during this phase. This is crucial to ensure that the platform does not perpetuate or exacerbate existing health disparities.Transitioning from existing, often fragmented, healthcare record systems to the proposed digital framework presents significant challenges. The pilot study should identify potential obstacles in data migration, data standardization, system interoperability, and the integration of the MDP with existing electronic health record (EHR) systems used in different Local Health Units across Italy. A phased approach to integration, potentially starting with a single Local Health Unit, may be necessary. Training healthcare professionals on the new system and addressing workflow changes will be essential. Potential barriers to adoption, such as varying levels of digital literacy among patients and caregivers, and lack of trust in AI-driven healthcare, must be proactively addressed. The pilot study should include measures to evaluate user acceptance and identify specific barriers to engagement, allowing for iterative improvements based on real-world feedback. Educational programs or support systems may be required to ensure seamless adoption.This protocol, while acknowledging GDPR compliance, needs a more thorough examination of potential AI bias to prevent exacerbating healthcare disparities. Addressing adoption barriers—including varying digital literacy and trust in AI—is also critical. The Salerno pilot study will address these issues by generating data on bias and adoption challenges. Ensuring equitable and effective AI requires a multi-faceted approach encompassing diverse training data, digital literacy education, transparent communication about AI capabilities and limitations, robust feedback mechanisms, human oversight, proactive audits, stakeholder engagement, and platform customization for diverse users. Community engagement and policy advocacy for equitable technology access are also vital. Specifically, the pilot will detail rigorous data anonymization, precise encryption methods (e.g., AES-256), robust access controls, clear data usage agreements, and bias mitigation strategies (e.g., balanced datasets, fairness-aware algorithms, regular audits) to ensure patient privacy, data security, and equitable care delivery.

## 5 Conclusions

The high prevalence of comorbidities among individuals with ASD emphasizes the critical need for a multidisciplinary approach to their care. A comprehensive, team-based approach allows healthcare providers to address these diverse needs holistically, involving specialists from different fields. By coordinating expertise across disciplines, providers can achieve a more accurate understanding of each patient’s unique profile, enabling them to tailor interventions that address both ASD symptoms and associated conditions. This collaborative model not only improves diagnosis and treatment effectiveness but also supports better long-term health outcomes, enhancing the overall quality of life for individuals with ASD and reducing the strain on caregivers.

## References

[1] American Psychiatric Association . Diagnostic and Statistical Manual of Mental Disorders. Fifth Edition. Arlington, VA: American Psychiatric Association (2013).

[2] Frith U Happé F . Autism spectrum disorder. Curr Biol. (2005) 15:R786–90. doi: 10.1016/j.cub.2005.09.033 16213805

[3] Hodges H Fealko C Neelkamal S . Autism spectrum disorder: definition, epidemiology, causes, and clinical evaluation. Transl Pediatr. (2020) 9:S55–65. doi: 10.21037/tp.2019.09.09 PMC7082249 32206584

[4] Kodak T Bergmann S . Autism spectrum disorder: characteristics, associated behaviors, and early intervention. Pediatr Clin North Am. (2020) 67:525–35. doi: 10.1016/j.pcl.2020.02.007 32443991

[5] Talantseva OI Romanova RS Shurdova EM Dolgorukova TA Sologub PS Titova OS . The global prevalence of autism spectrum disorder: A three-level meta-analysis. Front Psychiatry. (2023) 14:1071181. doi: 10.3389/fpsyt.2023.1071181 36846240 PMC9947250

[6] Scattoni ML Fatta LM Micai M Sali ME Bellomo M Salvitti T . Autism spectrum disorder prevalence in Italy: a nationwide study promoted by the Ministry of Health. Child Adolesc Psychiatry Ment Health. (2023) 17:125. doi: 10.1186/s13034-023-00673-0 37898807 PMC10613370

[7] Rylaarsdam L Guemez-Gamboa A . Genetic causes and modifiers of autism spectrum disorder. Front Cell Neurosci. (2019) 13:385. doi: 10.3389/fncel.2019.00385 31481879 PMC6710438

[8] Dietert RR Dietert JM Dewitt JC . Environmental risk factors for autism. Emerg Health Threats J. (2011) 4:7111. doi: 10.3402/ehtj.v4i0.7111 24149029 PMC3168222

[9] Cakir J Frye RE Walker SJ . The lifetime social cost of autism: 1990–2029. Res Autism Spectr Disord. (2020) 72:101502. doi: 10.1016/j.rasd.2019.101502

[10] Micai M Fulceri F Salvitti T Romano G Scattoni ML . Access and cost of services for autistic children and adults in Italy: a carers' perspective. Front Psychiatry. (2024) 15:1299473. doi: 10.3389/fpsyt.2024.1299473 38532989 PMC10963481

[11] Leader G Abberton C Cunningham S Gilmartin K Grudzien M Higgins E . Gastrointestinal symptoms in autism spectrum disorder: A systematic review. Nutrients. (2022) 14:1471. doi: 10.3390/nu14071471 35406084 PMC9003052

[12] Boulter C Freeston M South M Rodgers J . Intolerance of uncertainty as a framework for understanding anxiety in children and adolescents with autism spectrum disorders. J Autism Dev Disord. (2014) 44:1391–402. doi: 10.1007/s10803-013-2001-x 24272526

[13] Hudson CC Hall L Harkness KL . Prevalence of depressive disorders in individuals with autism spectrum disorder: a meta-analysis. J Abnorm Child Psychol. (2019) 47:165–175. doi: 10.1007/s10802-018-0402-1 29497980

[14] Craig F Lamanna AL Margari F Matera E Simone M Margari L . Overlap between autism spectrum disorders and attention deficit hyperactivity disorder: searching for distinctive/common clinical features. Autism Res. (2015) 8:328–37. doi: 10.1002/aur.1449 PMC4654237 25604000

[15] Ribolsi M Fiori Nastro F Pelle M Medici C Sacchetto S Lisi G . Recognizing psychosis in autism spectrum disorder. Front Psychiatry. (2022) 13:768586. doi: 10.3389/fpsyt.2022.768586 35295770 PMC8918655

[16] Riccioni A Siracusano M Vasta M Ribolsi M Nastro FF Gialloreti LE . Clinical profile and conversion rate to full psychosis in a prospective cohort study of youth affected by autism spectrum disorder and attenuated psychosis syndrome: A preliminary report. Front Psychiatry. (2022) 23:950888. doi: 10.3389/fpsyt.2022.950888 PMC9542639 36213900

[17] De Giorgi R De Crescenzo F D'Alò GL Rizzo Pesci N Di Franco V Sandini C . Prevalence of non-affective psychoses in individuals with autism spectrum disorders: A systematic review. J Clin Med. (2019) 8:1304. doi: 10.3390/jcm8091304 31450601 PMC6780908

[18] Souri A Hussien A Hoseyninezhad M Norouzi M . A systematic review of ioT communication strategies for an efficient smart environment. Trans Emerging Telecommunications Technol. (2019) 33:2161–3915. doi: 10.1002/ett.3736

[19] Pashazadeh A Navimipour NJ . Big data handling mechanisms in the healthcare applications: A comprehensive and systematic literature review. J BioMed Inform. (2018) 82:47–62. doi: 10.1016/j.jbi.2018.03.014 29655946

[20] Souri A Ghafour MY Ahmed AM Safara F Yamini A Hoseyninezhad M . A new machine learning-based healthcare monitoring model for student’s condition diagnosis in Internet of Things environment. Soft Comput. (2020) 24:17111–21. doi: 10.1007/s00500-020-05003-6

[21] Wohofsky L Scharf P Lattacher SL Krainer D . Assistive technology to support people with autism spectrum disorder in their autonomy and safety: A scoping review. Technology and Disability (2022) 34:1–11. doi: 10.3233/TAD-210355

[22] Hosseinzadeh M Koohpayehzadeh J Bali AO Rad FA Souri A Mazaherinezhad A . A review on diagnostic autism spectrum disorder approaches based on the Internet of Things and Machine Learning. J Supercomput. (2021) 3:2590–608. doi: 10.1007/s11227-020-03357-0

[23] Huang K Altosaar J Ranganath R . ClinicalBERT: modeling clinical notes and predicting hospital readmission. ArXiv. (2019). doi: 10.48550/arXiv.1904.05342

[24] Jungmann SM Klan T Kuhn S Jungmann F . Accuracy of a chatbot (Ada) in the diagnosis of mental disorders: comparative case study with lay and expert users. JMIR Form Res. (2019) 3:e13863. doi: 10.2196/13863 31663858 PMC6914276

[25] Schmidgall S Ziaei R Harris C Reis E Jopling J Moor M . AgentClinic: a multimodal agent benchmark to evaluate AI in simulated clinical environments. ArXiv. (2024). doi: 10.48550/arXiv.2405.07960

[26] Mehandru N Miao BY Almaraz ER Sushil M Butte AJ Alaa A . Evaluating large language models as agents in the clinic. NPJ Digit Med. (2024) 7:84. doi: 10.1038/s41746-024-01083-y 38570554 PMC10991271

[27] Rani A Yadav P Verma Y . Early-stage autism diagnosis using action videos and contrastive feature learning. Multimedia Syst. (2023) 29:2603–14. doi: 10.1007/s00530-023-01132-8

[28] Monaco F Vignapiano A Piacente M Pagano C Mancuso C Steardo L Jr . An advanced Artificial Intelligence platform for a personalised treatment of Eating Disorders. Front Psychiatry. (2024) 15:1414439. doi: 10.3389/fpsyt.2024.1414439 39165503 PMC11333353

[29] Pettorruso M Guidotti R d'Andrea G De Risio L D'Andrea A Chiappini S . Predicting outcome with Intranasal Esketamine treatment: A machine-learning, three-month study in Treatment-Resistant Depression (ESK-LEARNING). Psychiatry Res. (2023) :327:115378. doi: 10.1016/j.psychres.2023.115378 37574600

[30] Lord C Rutter M DiLavore PC Risi S Luyster RJ Gotham K . ADOS-2: autism diagnostic observation schedule. In: Edizione Italiana a Cura di Costanza C, 2nd ed. Hogrefe, Firenze, Italy. (2013).

[31] Klaiman C White S Richardson S McQueen E Walum H Aoki C . Expert clinician certainty in diagnosing autism spectrum disorder in 16-30-month-olds: A multi-site trial secondary analysis. J Autism Dev Disord. (2024) 54:393–408. doi: 10.1007/s10803-022-05812-8 36396807 PMC9672659

[32] Bishop SL Lord C . Commentary: Best practices and processes for assessment of autism spectrum disorder - the intended role of standardized diagnostic instruments. J Child Psychol Psychiatry. (2023) 64:834–8. doi: 10.1111/jcpp.13802 37005008

[33] Penner M Senman L Andoni L Dupuis A Anagnostou E Kao S . Concordance of diagnosis of autism spectrum disorder made by pediatricians vs a multidisciplinary specialist team. JAMA Netw Open. (2023) 6:e2252879. doi: 10.1001/jamanetworkopen.2022.52879 36696109 PMC10187485

[34] Lord C Rutter M Le Couteur A . Autism Diagnostic Interview-Revised: a revised version of a diagnostic interview for caregivers of individuals with possible pervasive developmental disorders. J Autism Dev Disord. (1994) 24:659–85. doi: 10.1007/BF02172145 7814313

[35] Orsini A Pezzuti L . Wechsler Intelligence Scale for Children: Manuale di Somministrazione e Scoring. In: Giunti Psychometrics, 4th ed. Firenze, Italy (2013), ISBN: 978-88-09-76995-3.

[36] Leiter RG Miller LJ . Leiter international performance scale. In: Leiter-3, 3rd ed. Nisonger Center Psychology, Ohio State University, Columbus, OH, USA (2013).

[37] Raven J Raven J . Progressive matrices. In: McCallum RS , editor. Handbook of Nonverbal Assessment. Springer US, Boston, MA, USA (2003), ISBN: 978-1-4613- 4945-7. p. 223–37.

[38] Griffiths R Battaglia FM . GMDS-R: griffiths mental development scales, revised: 0-2 anni: manuale/ruth griffiths. In: Giunti OS , editor. Edizione italiana a cura di Francesca Maria Battaglia e Margherita Savoini. Organizzazioni Speciali, Firenze, Italy (2007), ISBN: 978-88-09-40289-8. p. 1–137.

[39] Balboni G Belacchi C Bonichini S Coscarelli A Vineland II . Vineland adaptive behavior scales. In: Survey Form- Standardizzazione Italiana, 2nd ed. Giunti Psychometrics, Florence, Italy (2016), ISBN: 9788809994737. p. 1–317.

[40] Oakland T . Practical resources for the mental health professional. In: Adaptive Behavior Assessment System-II: Clinical Use and Interpretation; Elsevier: Amsterdam, The Netherlands. Academic Press, Heidelberg, Germany (2008). p. 1–410.

[41] Lee EE Torous J De Choudhury M Depp CA Graham SA Kim HC . Artificial intelligence for mental health care: clinical applications, barriers, facilitators, and artificial wisdom. Biol Psychiatry Cognit Neurosci Neuroimaging. (2021) 6:856–64. doi: 10.1016/j.bpsc.2021.02.001 PMC8349367 33571718

[42] Cui W Jeong W Thölke P Medani T Jerbi K Joshi AA . Neuro-GPT: developing a foundation model for EEG. ArXiv. (2023) 2311.03764:107.

[43] Liu Y Ma Y Zhou W Zhu G Zheng N . BrainCLIP: bridging brain and visual-linguistic representation via CLIP for generic natural visual stimulus decoding. (2023). doi: 10.48550/arXiv.2302.12971 40031248

[44] Li J Liu C Cheng S Arcucci R Hong S . Frozen language model helps ecg zero-shot learning. (2023). doi: 10.48550/arXiv.2303.12311

[45] Fucà E Guerrera S Valeri G Casula L Novello RL Menghini D . Psychiatric comorbidities in children and adolescents with high-functioning autism spectrum disorder: A study on prevalence, distribution and clinical features in an italian sample. J Clin Med. (2023) 12:677. doi: 10.3390/jcm12020677 36675606 PMC9864301

